# Assessment of Femoral Artery Bifurcation Level with Conventional Angiography

**DOI:** 10.7759/cureus.3479

**Published:** 2018-10-22

**Authors:** Muhammad N Mengal, Tariq Ashraf, Syed N Hassan Rizvi, Abida Badini, Musa Karim

**Affiliations:** 1 Cardiology, National Institute of Cardiovascular Diseases (NICVD), Karachi, PAK; 2 Family Medicine, The Aga Khan University, Karachi, PAK; 3 Miscellaneous, National Institute of Cardiovascular Diseases (NICVD), Karachi, PAK

**Keywords:** femoral artery, coronary angiography, femur head, punctures, aneurysm, fistula

## Abstract

Introduction

The common femoral artery (CFA) is the optimal access point for femoral arterial puncture. A higher or lower puncture can result in various vascular complications and by the proper definition of the femoral arterial bifurcation level and the optimal puncture point such complications can potentially be avoided. In the literature, little data is available about the frequency of femoral artery bifurcation and the relationship between the bifurcation level of one artery and its contralateral counterpart in our part of the world.

Methods

We performed a prospective study from April 2016 to September 2016 to define the frequency of bifurcation of the CFA in relation to the femoral head and the relationship between bilateral CFA bifurcations, with bilateral femoral angiography on 579 patients undergoing routine coronary angiography.

Results

The frequency of normal/low, high, and very high femoral bifurcations was 66%, 26%, and 8%, respectively. There was no significant difference in the bifurcation of CFA between the two sides (p = 0.51). A specific bifurcation level on one side significantly increased the likelihood of the same bifurcation level on the contralateral side (odds ratio (OR) = 151.86 (51.39-448.77)). A multivariable logistic regression analysis revealed age, race, gender, height and weight, body surface area (BSA), and body mass index (BMI) were not predictive of any specific bifurcation level on either side.

Conclusions

The majority (two-thirds) of the individuals in the study population were with normal/low femoral bifurcation with no significant difference in bifurcation level on either side.

## Introduction

Advances in interventional technology have facilitated the emergence of percutaneous procedures requiring large-caliber sheaths and bilateral femoral arterial access [1].

Common femoral artery (CFA) is the frequently used access site because of being of relatively large caliber, superficial, and fixed. It courses over the femoral head [2] and in the majority of patients, the bifurcation is below the level of the mid-femoral head [3]. This relationship of the artery to bone allows us easy manual arterial compression and plays a role in reducing the risk of prolonged bleeding after the removal of the catheter or sheath [4].

In both antegrade and retrograde punctures, the optimal technique is a single anterior wall puncture well above the bifurcation and below the origin of the inferior epigastric artery [5].

Vascular complications are more common when access is not in the common femoral arterial segment. Knowledge of the anatomical relationship of the femoral artery to the femoral head helps the operator avoid vascular complications. When bilateral femoral arterial access is required, knowledge of the relationship between the right and left femoral artery bifurcation levels may help the operator predict the bifurcation level on one side based on the level of bifurcation on the contralateral side. There is little data in the literature to help and guide the operators in this respect. If the puncture site is proximal, there is a risk of entering the external iliac artery, increasing the risk of retroperitoneal hemorrhage. While a distal puncture can result in entering one of the two branches of the CFA (profunda femoris or superficial femoral artery) and with the risk of vascular complications like a pseudoaneurysm or an arteriovenous fistula [5-10]. Due to interpersonal variations in anatomy, the high variability of the CFA bifurcation level with respect to the femoral head can result in an entry point into the artery that is either too low (below the bifurcation) or too high (in the external iliac artery), even though the puncture site in relation to the femoral head may be in the desired zone [3]. It can result in an access location unsuitable for the serial dilatation and insertion of a large-caliber sheath in advanced procedures. We, therefore, analyzed the frequency of femoral artery bifurcation on both sides, compared its bifurcation level in relationship to the femoral head on each side with bilateral femoral angiograms in our local patient population. We then compared the bifurcation level on both sides and described the likelihood of a specific bifurcation level on one side that may increase the likelihood of a bifurcation level on the contralateral side.

## Materials and methods

We are a high-volume tertiary care center performing about 17,000 to 18,000 cases a year. Nearly half of our cases are diagnostic coronary angiograms. We selected a sample size (using the Raosoft system, Raosoft, Inc., Seattle, WA, US) of 579 patients (from 4500 patients, keeping the margin of error as 5% and with a confidence level of 99%) of either sex, aged 20-80 years for femoral angiograms who were undergoing left heart catheterization from April 2016 to September 2016. Patients with chronic kidney disease (CKD), cardiogenic shock, LM ± 3VCAD, and severe left ventricular (LV) dysfunction were excluded from the study to avoid an excessive contrast agent in such high-risk patients. After routine coronary angiography with informed consent and in the absence of exclusion criteria, all the patients underwent a bilateral femoral angiography. For the left femoral artery angiogram, we used the JR4 (Medtronic) diagnostic catheter and took an angiogram with a hand-held injection in the left anterior oblique (LAO) degree 20 projection. For the right femoral artery, we gave a hand-held injection through the 6 French access sheath and recorded the angiogram in right anterior oblique (RAO) degree 20 projections. The femoral angiograms of these patients were examined for the level of femoral artery bifurcation with respect to the femoral head and compared bilaterally. Our study was approved by the ethical review committee of the institution (ERC # 04/2016).

**Figure 1:**
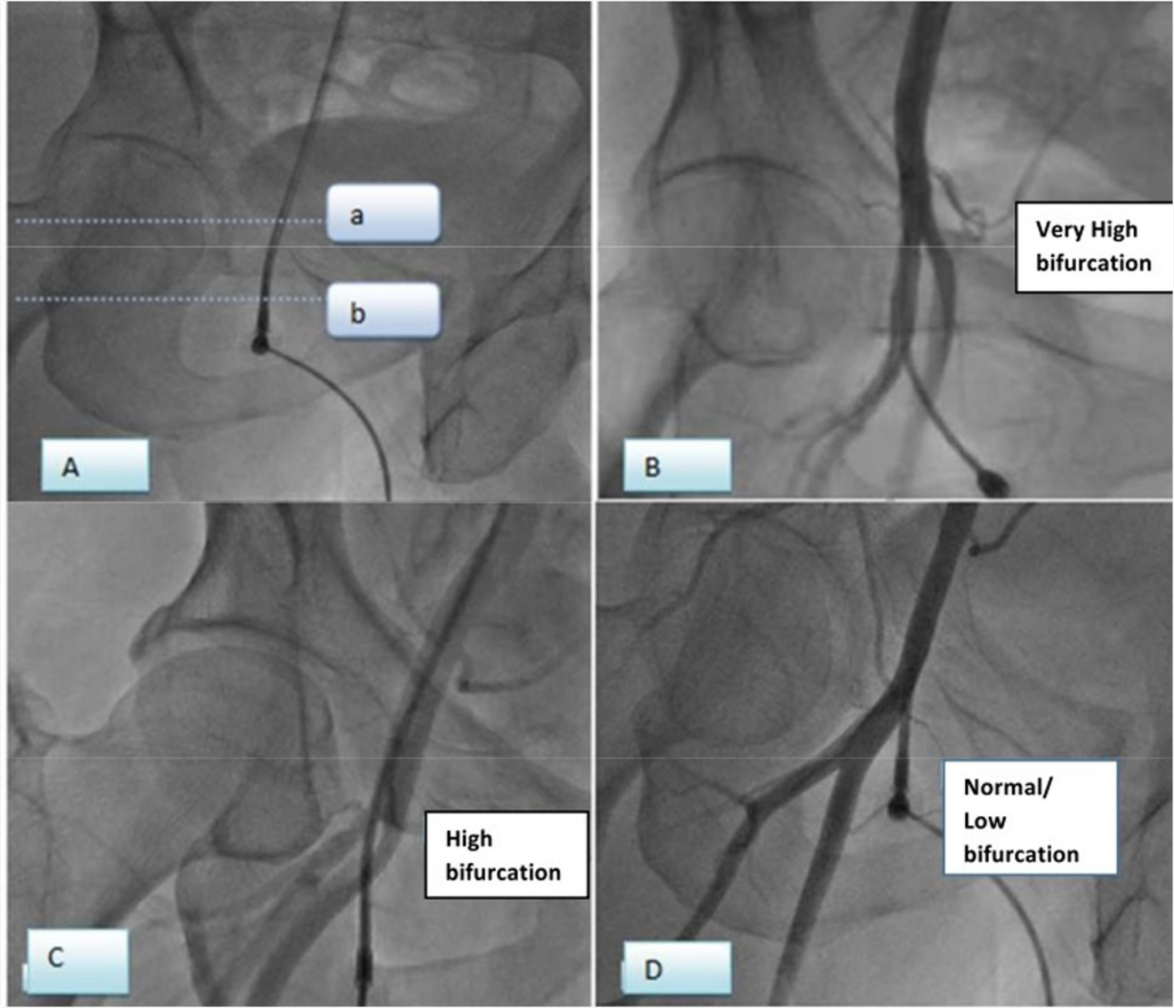
Bifurcation level of the femoral artery Figure 1A defining the femoral artery bifurcation level: Bifurcation above line a is defined as Very High, between the lines a and b is defined as High and below line b is Normal/Low bifurcation (femoral arterial sheath can also be seen). Figure 1B, Figure 1C, and Figure 1D showing femoral artery bifurcation level; 1B Very High, 1C High, and 1D Normal/Low

The common femoral artery (CFA) is the main arterial supply of the lower limb. It courses down to the leg as the continuation of the external iliac artery behind the inguinal ligament and divides in the thigh into two branches: the profunda femoral artery (PFA) and the superficial femoral artery (SFA). We took the femoral head as the radiologic landmark for defining the level of the femoral artery bifurcation. Femoral artery bifurcation was assigned one of three designations in relation to the femoral head, as shown in Figure 1A. When the bifurcation level of the femoral artery was inferior to the inferior border of the femoral head, the bifurcation level of the femoral artery was considered a “normal/low” level of bifurcation (Figure 1D). When the bifurcation level was superior to the inferior border of the femoral head but inferior to the midpoint of the femoral head, it was considered a “high” bifurcation of the femoral artery (Figure 1C). And when the bifurcation level was superior to the midpoint of the femoral head, the bifurcation of the femoral artery was assigned as a “very high” bifurcation (Figure 1B). One of our patients had a hip replacement with a prosthesis; three of our senior cardiologists reviewed the level of bifurcation and came to a consensus. These landmarks are different from those used for clinical purposes [11], but these are the most useful and well-utilized landmarks of the femoral head for the definition of the femoral arterial relationship anatomically.

As mentioned above, femoral angiograms were recorded in the 20° ipsilateral anterior oblique view with no cranial or caudal angulation and were reviewed for the determination of the level of femoral artery bifurcation with respect to the femoral head. The bilateral femoral artery angiogram comparison was recorded. Various demographic variables like age, gender, race, height, weight, body surface area (BSA), and body mass index (BMI), which could plausibly be associated with variations in the site of bifurcation were collected for review.

Data were analyzed for pre-specified endpoints. A multivariate logistic regression analysis was performed to evaluate the association between demographic and angiographic variables with the level of femoral artery bifurcation. All statistical analyses were performed using IBM SPSS Statistics for Windows, Version 21.0 (IBM Corp., Armonk, NY, US).

## Results

We performed a total of 579 femoral angiograms. The baseline characteristics of these patients are listed in Table 1.

**Table 1:** Baseline characteristics

Characteristics	Frequency (%) or Mean ± SD
Age (yrs.)	56.24±9.9
Male	388 (67.01%)
Female	191 (32.98%)
Height (cm)	159.24 ± 8.5
Weight (Kg)	67.63 ± 9.9
Body Mass Index (kg/meter^2^)	26.7 ± 3.3
Body Surface Area (meter^2^)	1.72 ± 0.4

There were 388 (67.01) % males and 191 (32.98%) females, with a mean age of 56.24 ± 9.9. The number of patients with normal/low, high, and very high bifurcations of the left common femoral artery was 372 (64.2%), 154 (26.5%), and 53 (9.1%), respectively. The number of patients with normal/low, high, and very high bifurcations of the right common femoral artery was 389 (67.1%), 148 (25.5%), and 42 (7.25%), respectively (Table 2).

**Table 2:** Femoral artery bifurcation level in study population

Bifurcation Level	Frequency		Percent		P-value
	Left FA (n)	Right FA (n)	Left FA %	Right FA %	
Normal/Low	372	389	64.2	67.1	0.48
High	154	148	26.5	25.5	0.75
Very High	53	42	9.1	7.25	0.49
Total	220	220	100	100	

No statistically significant difference was noted in the proportion of patients with normal/low, high, or very high bifurcations on the left as compared to the right (p=0.51). Another important observation was the presence of a bifurcation concordance between the two sides so that a normal/low, high, and very high bifurcation on either side increases the likelihood of a high and very high bifurcation on the contralateral side (Chi-square test p-value <0.01). If either of the common femoral arteries bifurcates high, the odds ratio (OR) of the contralateral common femoral artery to bifurcate high is 151.86 (95% confidence interval (CI) 51.39-448.77). Furthermore, a multivariable logistic regression analysis showed that age, gender, height, weight, body mass index, and body surface area were not predictive of any specific bifurcations on either side.

## Discussion

The common femoral artery is the most commonly accessed artery for performing both cardiac and peripheral percutaneous vascular procedures. Femoral artery vascular access complications are very high, being in the range of 2%-10% [1]. Our study showed that almost 35% of patients will have a high bifurcation in either of their femoral arteries. And if one of the femoral arteries has a high or very high bifurcation, the contralateral femoral artery is more likely to have a high bifurcation. Furthermore, the prevalence of normal/low, high, and very high bifurcations does not differ between the right and left, so choosing one side over the other will not reduce the risk of encountering a high bifurcation. This information regarding bifurcation on one side will assist the operator in planning contralateral femoral artery access. The rates of high or very high femoral bifurcations appear high at first glance but our data are consistent with prior literature, in particular, the study by Gupta et al. [1] in UCSF and that by Schnyder et al. [3] both showed almost similar results, as shown in Table 3.

**Table 3:** Comparison of our study at NICVD with other studies NICVD: National Institute of Cardiovascular Diseases; UCSF: University of California, San Francisco

Level of femoral artery bifurcation	NICVD	UCSF [1]	Schnyder et al. [3]
Normal/Low	66%	70%	56%
High	26%	26%	39%
Very High	8%	4%	5%

For the definition of common femoral artery bifurcation level, we chose a definition that was previously used in the literature and has an easy, practical applicability. We did not find any clinical/physical/anatomical predictors of CFA bifurcation before an initial access is attempted. There was no significant difference in the prevalence of a normal/low, high, or very high bifurcation on the left and right sides (p-value = 0.51).

As the femoral artery is the preferred arterial access route for major interventional procedures, defining its bifurcation level can invariably decrease the vascular complications in procedures like transcatheter aortic valve implantation (TAVI). In TAVI, most of the morbidity and mortalities are due to vascular complications. Every year, thousands of new candidates of TAVI are being added [11]. As the prevalence of patients requiring these percutaneous procedures increases, the need for bilateral femoral arterial access will also increase. Chronic total occlusion percutaneous coronary intervention (CTO PCI) procedures frequently require bilateral femoral artery access. The prevalence of CTOs is as high as 89% and 15% in patients with and without a prior history of coronary artery bypass graft surgery; the potential growth of CTO PCI is large [12]. CTOs account for 5%-10% of the total PCIs performed [13]. Our study will help the operator in planning contralateral femoral artery access if faced with a high femoral artery bifurcation on any one side. In one of the studies, it was found that routine ultrasound guidance improves the cannulation of CFA only in patients with high CFA bifurcations [14]. In such situations, ultrasound guidance can help in visualizing the femoral arterial bifurcation and that can then be correlated to the femoral head anatomically during angiography. Interventional procedures like percutaneous left ventricular support device placement and TAVI require the placement of a large-sized cannula in the femoral arteries. As mentioned in these procedures, the overall success is also determined by the safety of femoral arterial access. If the CFA puncture site is below the bifurcation of the artery, the size of the artery will not only limit the placement of large sheaths but with increased chances of aneurysms and pseudoaneurysms. Most of the vascular closure devices approved by FDA are for use in CFA. These devices have limited use in the branches of CFA if the access site is in a branch vessel [1].

Study limitations

We used definite bony anatomical landmarks of the femoral head to define the levels of bifurcation of the common femoral artery and our experienced interventional operators were used to define the bifurcation levels. Subjective errors could still not be completely avoided. We did not use Doppler ultrasound for evaluating the femoral artery bifurcation and comparing it with conventional angiography.

## Conclusions

This study is the first of its kind in our population defining the CFA bifurcation level. Our study showed that two-thirds of the individuals in our local population have a normal/low femoral bifurcation and one-third have a high bifurcation level with no significant difference in the bifurcation level on either side. Bifurcation of the common femoral artery at a specific level on one side increases the likelihood that the contralateral femoral artery will also have the same specific bifurcation level. In complex interventional procedures, this data can potentially help interventional cardiologists in planning the access site when bilateral femoral arterial access is required.
